# Supplementary material for: Resilient Antarctic soil bacteria consume trace gases across wide temperature ranges
Source: ISME J. 2026 Feb 10;20(1):wrag020. doi: 10.1093/ismejo/wrag020 (PMC12998438; doi:10.1093/ismejo/wrag020)
Supplement: Hutchinson_and_Holland-Supplementary_Info-Revised_2026_wrag020 [file hutchinson_and_holland-supplementary_info-revised_2026_wrag020.pdf]

Supplementary Information: Resilient Antarctic soil bacteria consume trace gases across wide temperature ranges

Supplementary Text

Generalised linear models to predict trace gas oxidation rates from temperature data

To investigate the effect of temperature on rates of trace gas oxidation, zero-inflated generalised linear models (GLM) with Gamma error distributions and log-link functions were fitted to bulk soil H_2, CO and CH_4 oxidation rates (Fig. S2, S11). Temperature was included as a second-degree polynomial covariate to allow for non-linear responses. For H_2, both polynomial terms in the conditional model were significant ($P = 2.07 \times 10^{-7}$ and $P < 2 \times 10^{-16}$, respectively), although effect sizes were small. The first- and second-order terms corresponded to multiplicative increases of $e^{-11.0894} = 1.53 \times 10^{-5}$ [95% CI 2.32×10^{-7}, 1.00×10^{-3}] and $e^{-19.3284} = 4.03 \times 10^{-9}$ [95% CI 9.00×10^{-11}, 1.80×10^{-7}] nmol H_2 h^{-1} g^{-1} for every 1°C temperature increase, respectively. Effects were stronger in the zero-inflation model, with temperature exerting a significant effect on the presence of H_2 oxidation, with estimates of $e^{25.6874} = 1.43 \times 10^{11}$ [95% CI 9.73×10^{6}, 2.10×10^{15}] and $e^{20.7044} = 9.81 \times 10^{8}$ [95% CI 2.97×10^{4}, 3.25×10^{13}] for the linear and quadratic terms, respectively. This means that there was a small but significant effect of temperature on both the likelihood and rate of H_2 consumption. H_2 oxidation rates ranged from 0.0001 to 8.65 nmol H_2 h^{-1} g^{-1} between -20 to 75°C, with the predicted peak rate occurring at 25°C (Fig. S11A). The greatest changes in the rate of hydrogen oxidation occurred over the temperature ranges of 1-13°C and 35-46°C. Model performance was significantly better than the null model ($P = 3.67 \times 10^{-10}$, chi-squared test on the difference of log likelihoods).

Rates of CO oxidation were also affected by temperature, though only the quadratic term of the conditional model was significant ($P = 8.7 \times 10^{-5}$). Effect sizes were again small, with multiplicative changes of $e^{-8.7058} = 1.66 \times 10^{-4}$ [95% CI 1.68×10^{-10}, 1.63×10^{2}] and $e^{-20.6470} = 1.08 \times 10^{-9}$ [95% CI 3.59×10^{-14}, 3.25×10^{-5}] for the first- and second-order term, respectively. The zero-inflation model showed a strong and significant temperature effect (estimates of $e^{85.2661} = 1.07 \times 10^{37}$ [95% CI 2.05×10^{21}, 5.61×10^{52}] and $e^{60.4947} = 1.87 \times 10^{26}$ [95% CI 4.56×10^{15}, 7.68×10^{36}] for the linear and quadratic terms, respectively. CO oxidation rates were lower than those for H_2, ranging from 2×10^{-5} to 0.028 nmol CO hr^{-1} g^{-1} (Fig. S11B), with a predicted peak at 37°C and the greatest rate changes between 4-15°C and 31-43°C. Model performance was significantly better than the null model ($P = 2.28 \times 10^{-6}$, chi-squared test on the difference of log likelihoods). Model prediction accuracy had a much lower mean squared error (MSE) of 1.79×10^{-5} compared to 2.07 for H_2.

In contrast, CH_4 oxidation showed no significant temperature dependence in the conditional component of the model; neither the linear nor quadratic term was significant ($P = 0.27$ and $P = 0.15$). CH_4 oxidation rates ranged from 1.8×10^{-4} to 0.06 nmol CH_4 h^{-1} g^{-1}, with no clear trend across temperatures (Fig. S11C). Only the zero-inflated component exhibited significant temperature effects (i.e. temperature only significantly affected the likelihood of CH_4 oxidation), and the full model did not outperform the null model ($P = 0.29$). Together, these analyses show that temperature strongly influences both the presence and magnitude of H_2 and CO oxidation but has no detectable effect on CH_4 oxidation rates within the measured range.

Supplementary Tables

Supplementary Table 1 (xlsx) - Sample metadata.

Supplementary Table 2 (xlsx) - Trace gas oxidation rates for Dronning Maud Land *in situ* and *ex situ* incubations.

Supplementary Table 3 (xlsx) - Microbial community metabolic marker genes (from short-read metagenomic data).

Supplementary Table 4 (xlsx) - MAG quality, taxonomy, and metabolic genes.

Supplementary Table 5 (xlsx) - Microbial community composition (from short-read metagenomic data).

Supplementary Table 6 (xlsx) – DRAM pathway results for aerotrophy processes.

Supplementary Table 7 (xlsx) - Trace gas oxidation rates for Robinson Ridge, Dronning Maud Land, and Bunger Hills soil samples across 14 temperatures, plus Arrhenius and GTD-modelled values.

Supplementary Table 8 (xlsx) - Comparative analyses of modelled oxidation rates in the future under different emissions scenarios.

Supplementary Figures

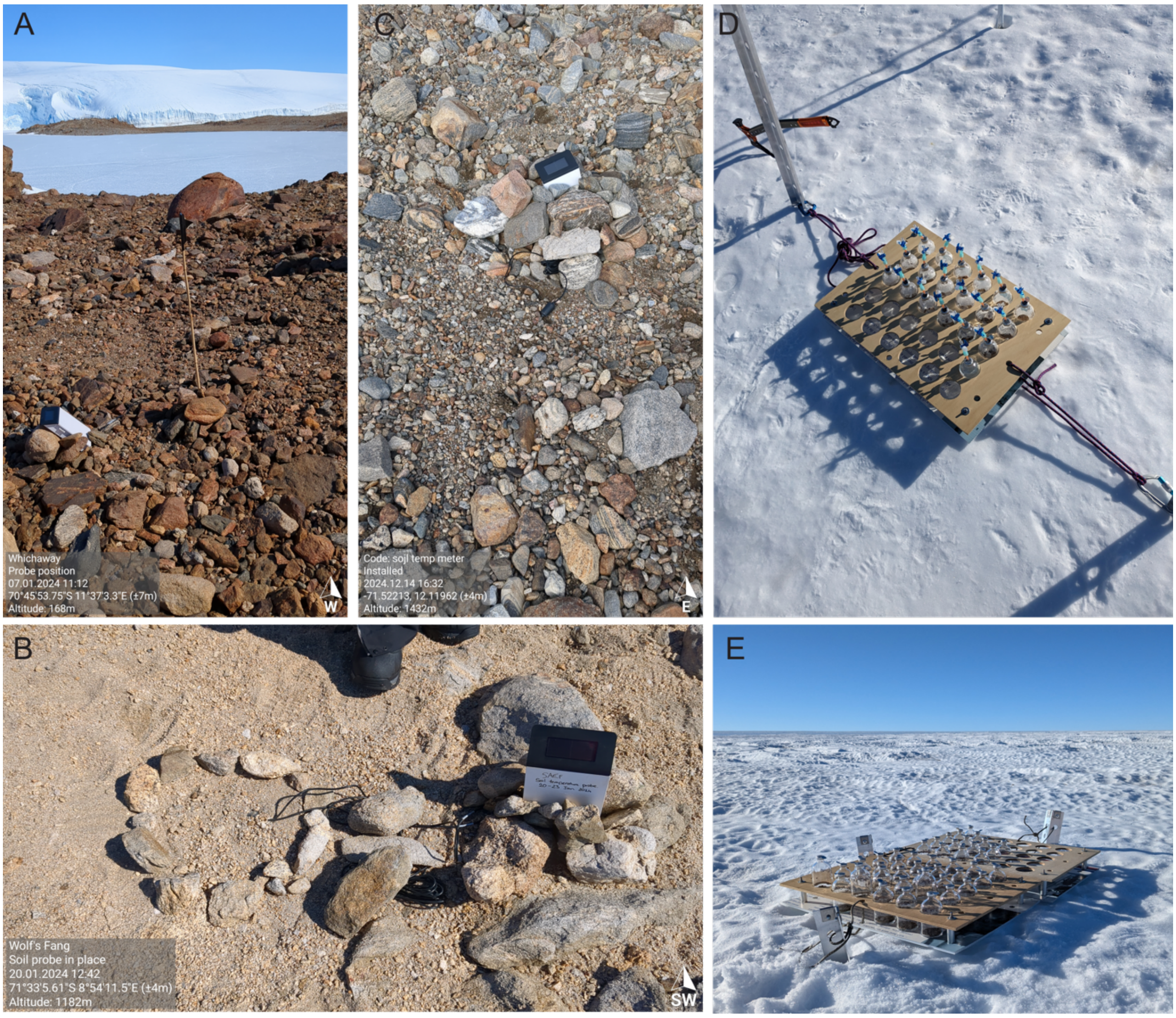

Figure S1. **Soil temperature sensor and *in situ* incubation locations.** Locations where soil temperature was recorded at 10 cm depth in (A) Schirmacher Oasis, January 2024, (B) Henriksen Nunataks, January 2024, and (C) Petermann Ranges, December 2024. *In situ* incubations of Antarctic soil microcosms at field camps in Dronning Maud Land in (D) January 2024 and (E) December 2024.

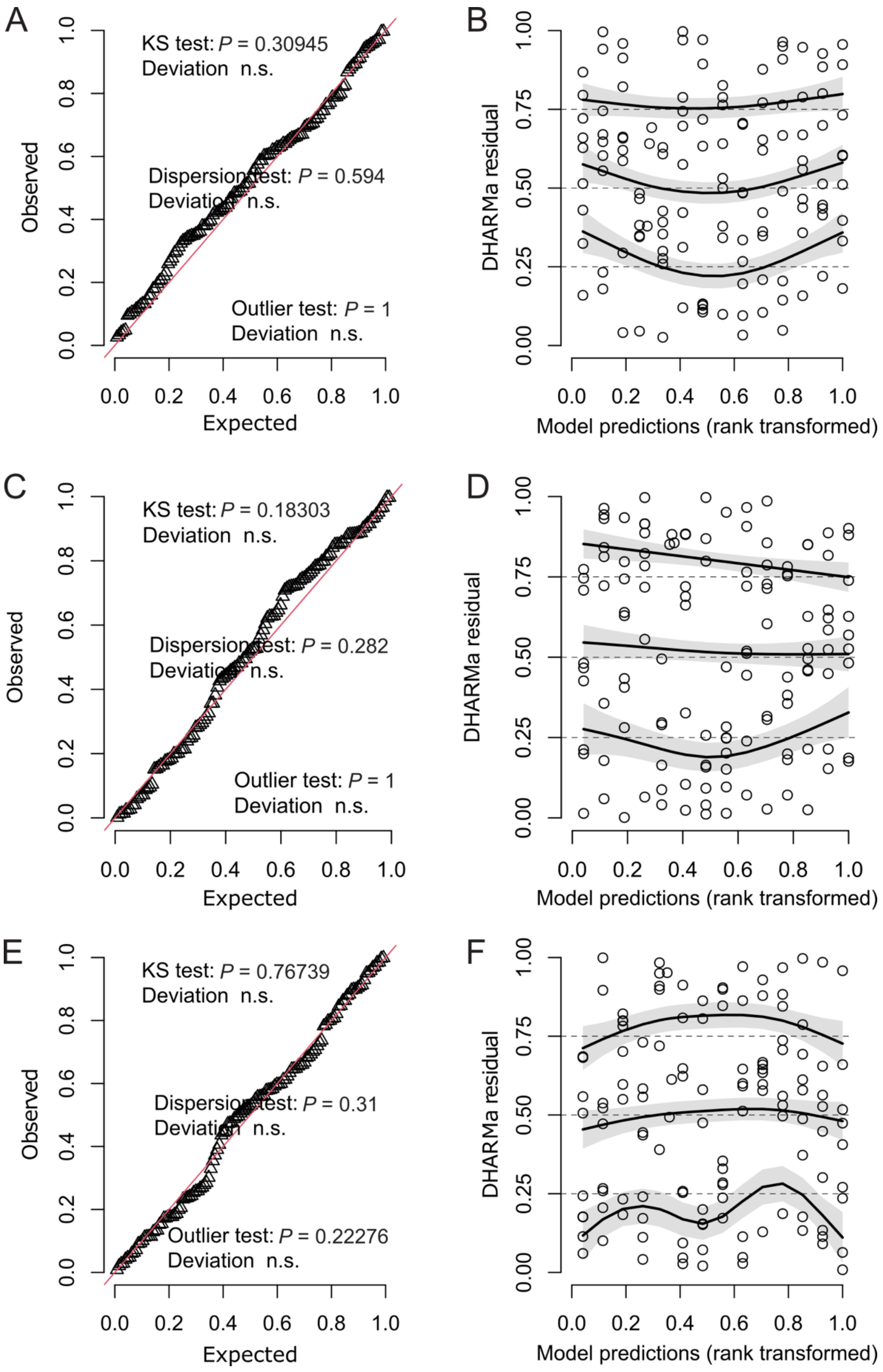

Figure S2. Assessment of model fit and diagnostics. QQ plot residuals for (**A**) H_2, (**C**) CO, and (**E**) CH_4. KS = DHARMa residual vs. predicted for (**B**) H_2, (**D**) CO, and (**E**) CH_4. No significant problems were detected in any of the residual vs. predicted plots.

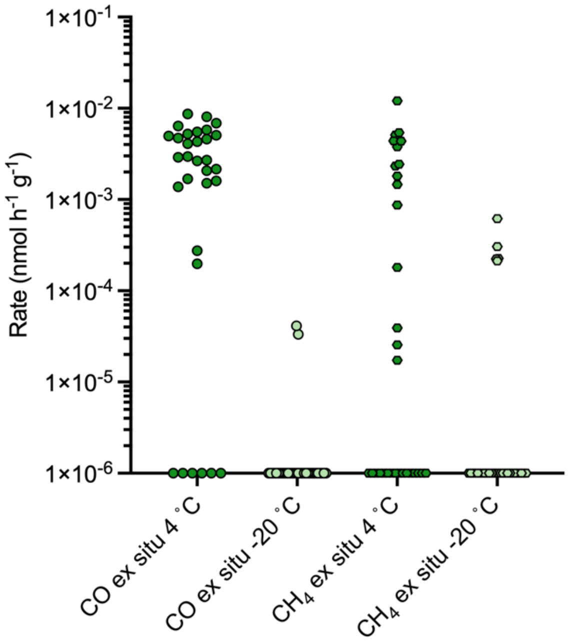

Figure S3. CO and CH_4 oxidation rates in *ex situ* microcosms of soils from Dronning Maud Land, Antarctica, incubated at 4 and -20°C (*n* = 31). Samples with a no observable trace gas consumption (i.e. a rate of 0) are nominally shown at 1×10^{-6} for representation on the log scale.

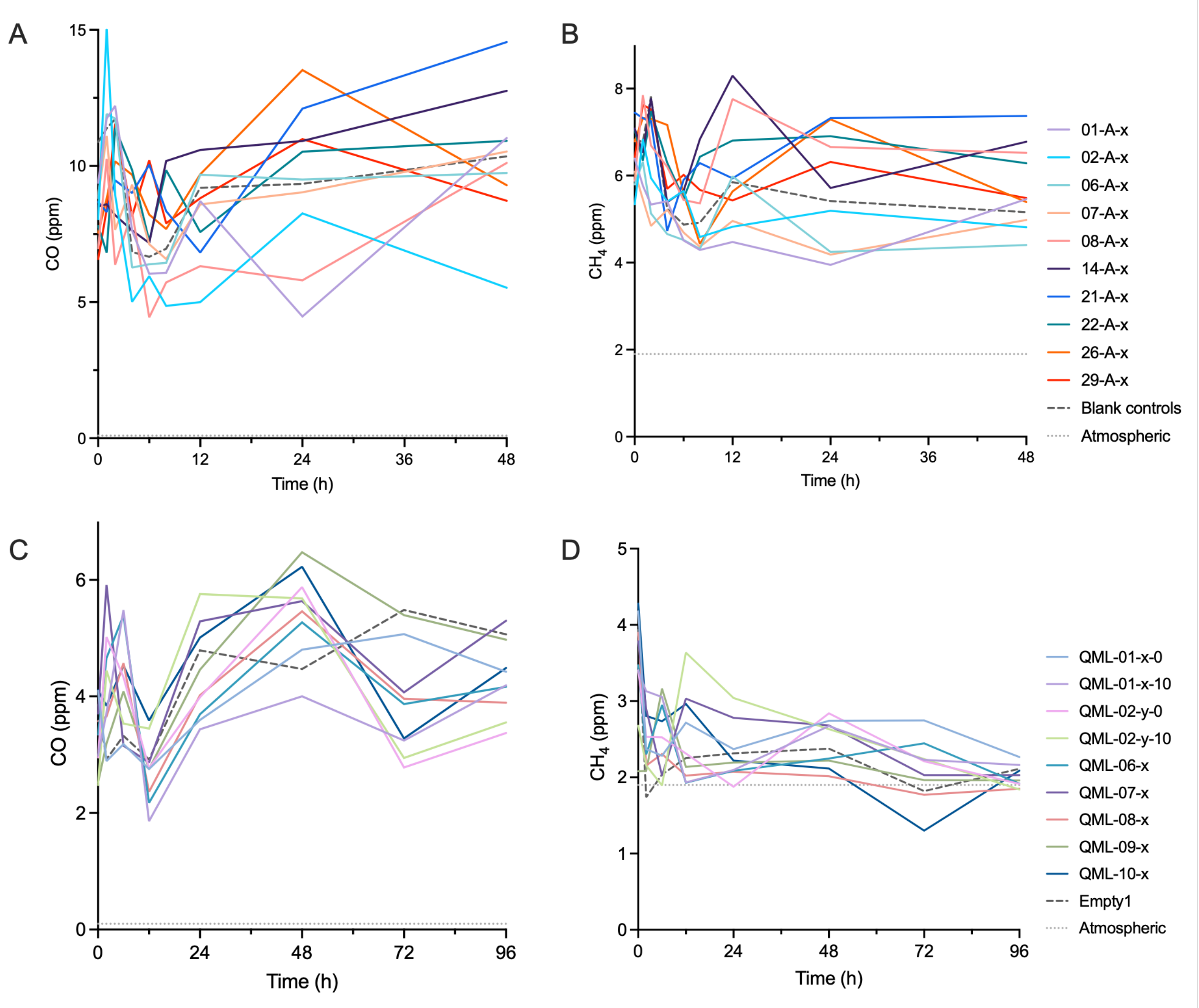

Figure S4. *In situ* incubations did not show conclusive CO or CH_4 consumption. (A) CO and (B) CH_4 measurements in 2023-24. (C) CO and (D) CH_4 measurements in 2024-25. All measured data points are triplicate averages. Error bars are not shown. Atmospheric concentration is also shown on each plot: 0.1 ppm for CO and 1.9 ppm for CH_4.

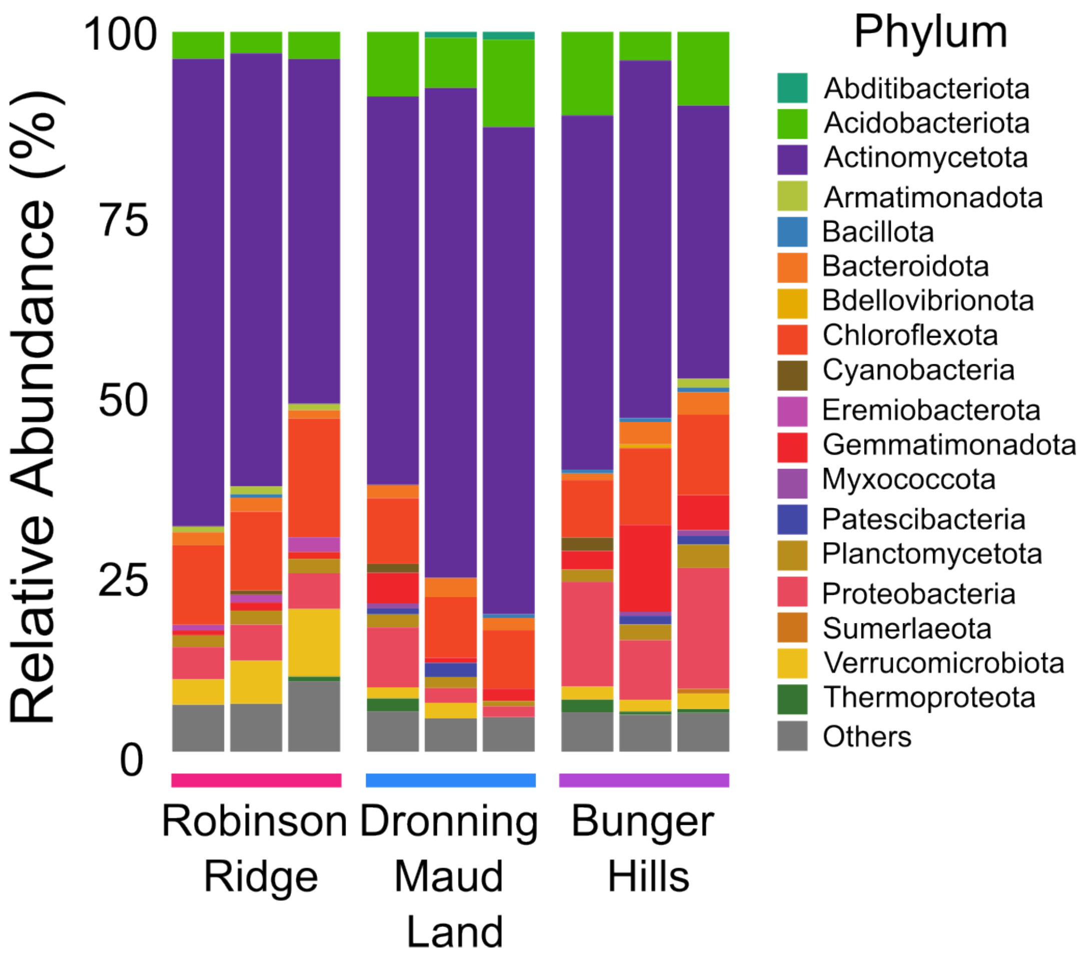

Figure S5. Taxonomic bar chart based on phyloFlash 16S rRNA gene taxonomic classification of the metagenomes. All phyla are bacterial except for Thermoproteota, which is archaeal. “Others” includes any bacterial and archaeal phyla with ≤100 counts.

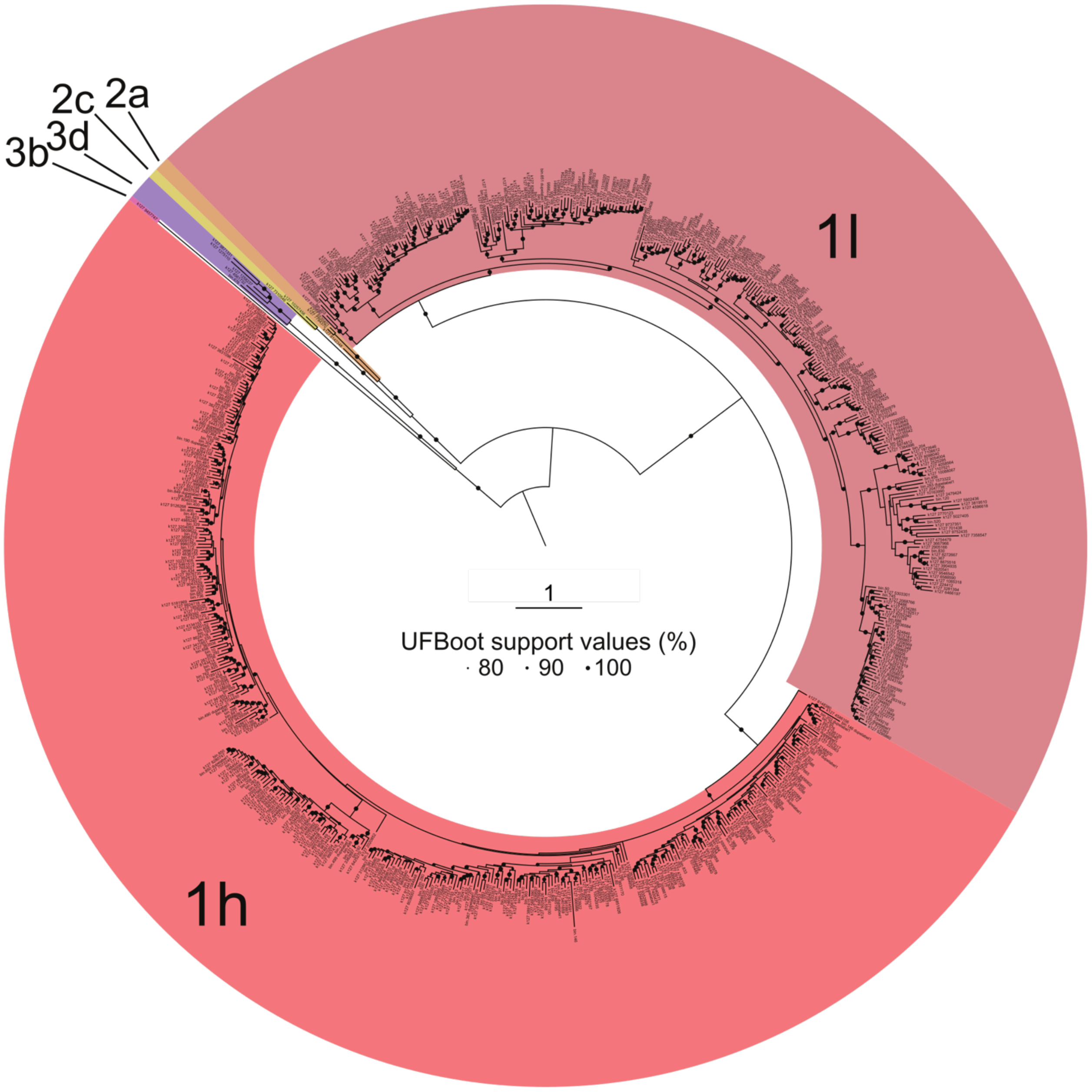

NiFe-Hydrogenases 1h, 1l, 2a: high-affinity H_2 oxidation
NiFe-Hydrogenases 2c: H_2 sensing
NiFe-Hydrogenases 3b, 3d: Hydrogenogenic fermentation (bidirectional)

Figure S6. Phylogenetic tree of all predicted [NiFe] hydrogenase proteins encoded in the metagenome. The hydrogenases group according to their predicted subgroups as predicted by metabolic marker gene annotation. Scale bar represents 1 nucleotide substitution.

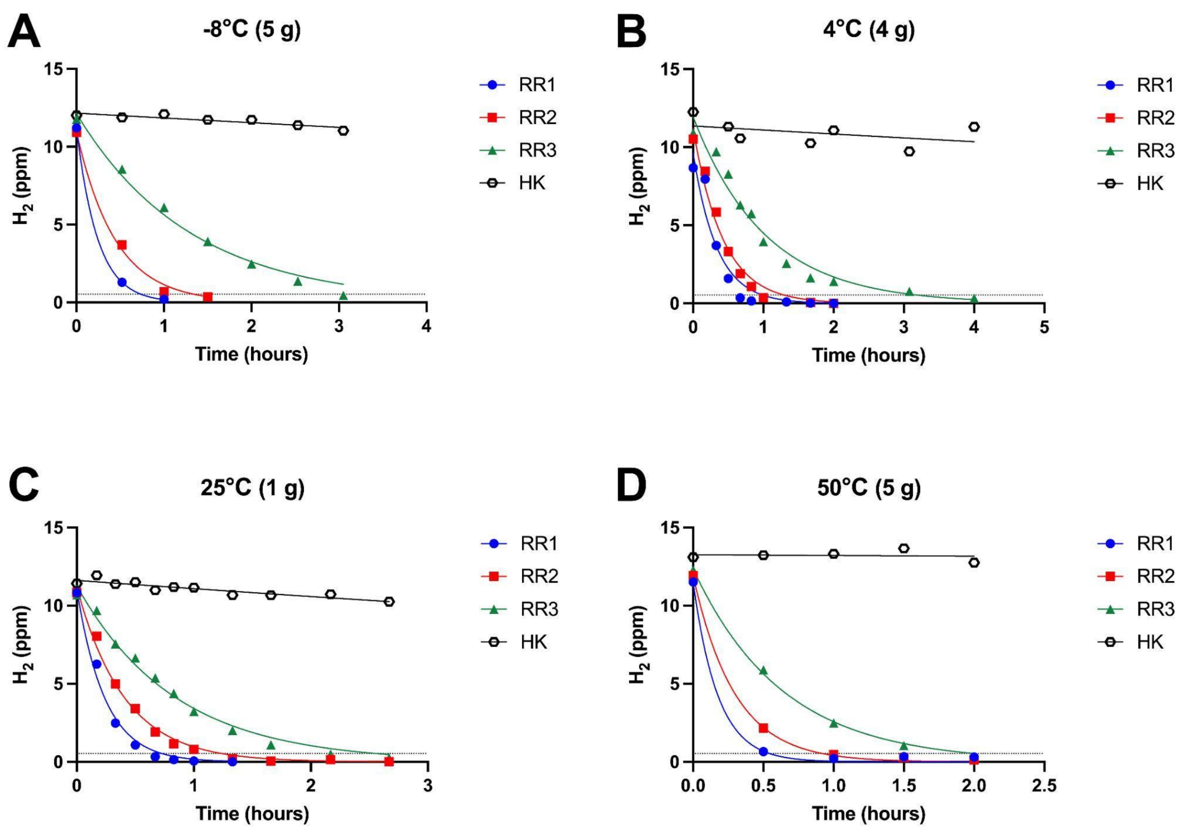

Figure S7. Antarctic soils consume H_2 to sub-atmospheric concentrations in short time scales. Select Robinson Ridge soils (RR1, RR2, RR3) and heat-killed controls incubated at (**A**) -8°C, (**B**) 4°C, (**C**) 25°C and (**D**) 50°C, showing H_2 oxidation to sub-atmospheric concentrations in less than 1 hour. The mass of soil used in each experiment is written in brackets in the graph title. Concentrations were fitted with non-linear regression models to derive rates. Dashed line indicates atmospheric H_2 concentration (0.53 ppm).

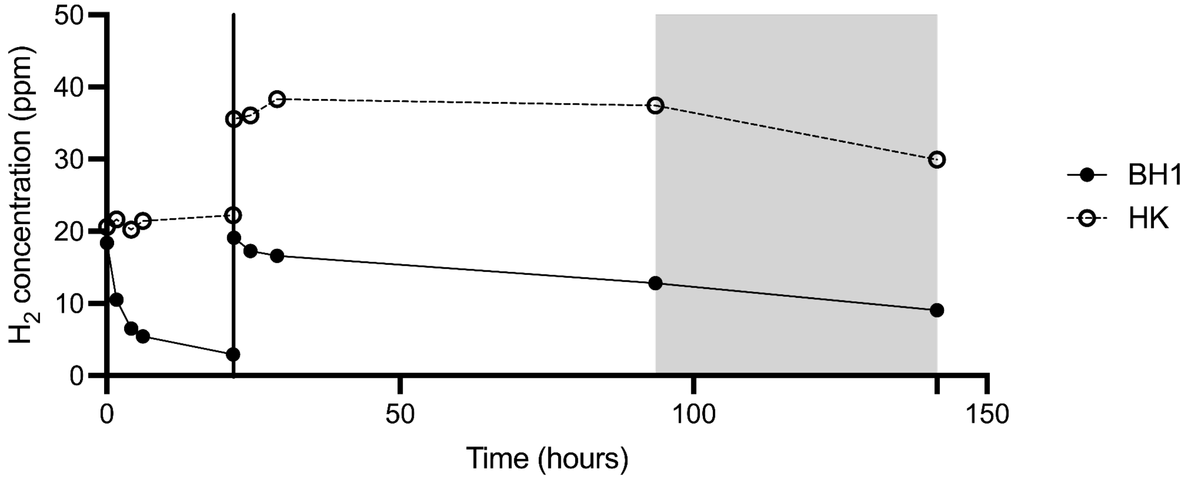

99

100 **Figure S8. Initial H_2 consumption by a Bunger Hills soil sample at 75°C, before inactivity.**
101 Bunger Hills (BH1) and heat-killed control (HK) soils at 75°C. Line at 24 h indicates injection of
102 another ~20 ppm H_2. Shaded region represents the microcosm returning to room temperature
103 (20°C).

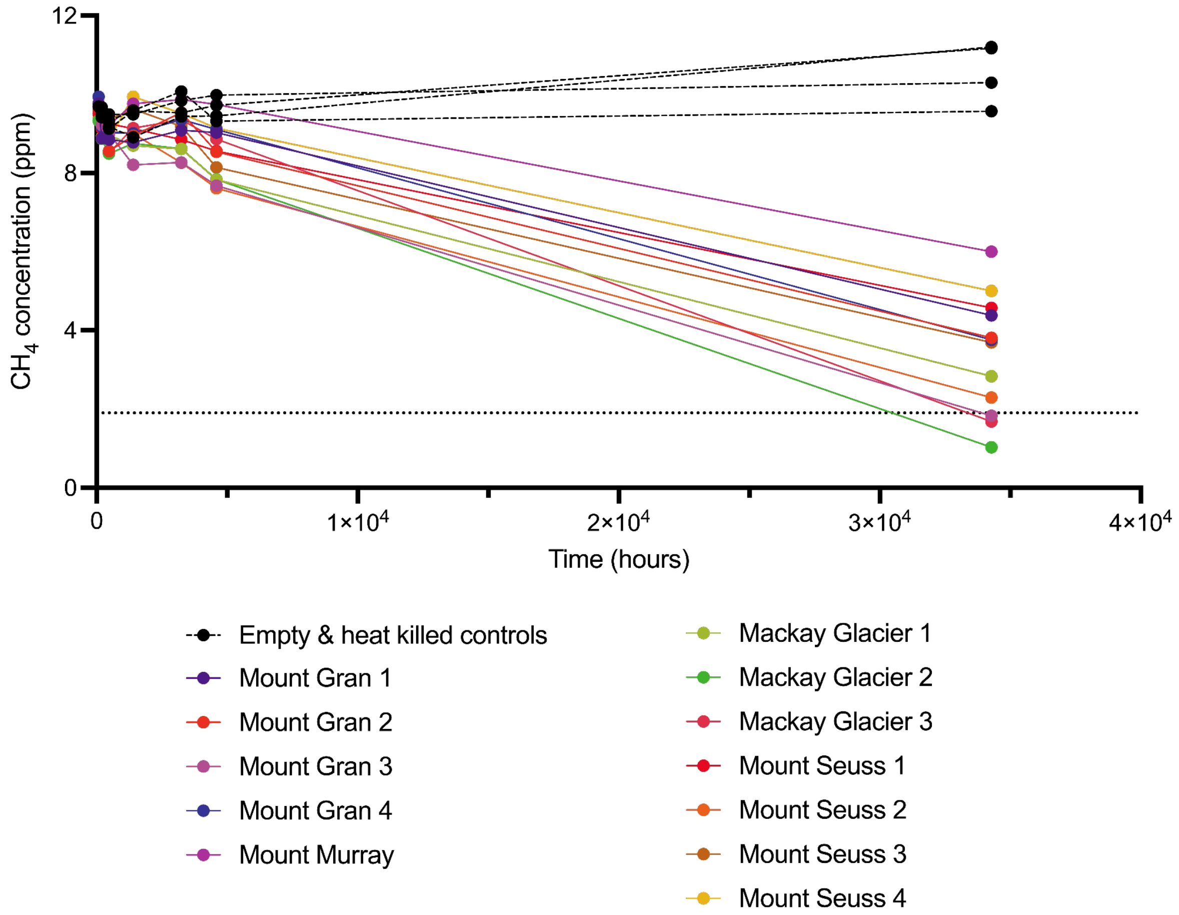

Figure S9. Methane (CH_4) oxidation at -20°C recorded after multiple years. Mackay Glacier soils and controls from Ortiz *et al.* (2021) were continuously incubated at -20°C. Data collected in the first ~6 months was published previously, while the final sampling occurred after ~4 years (34,273 h). Dotted line represents atmospheric CH_4 concentration (1.9 ppm).

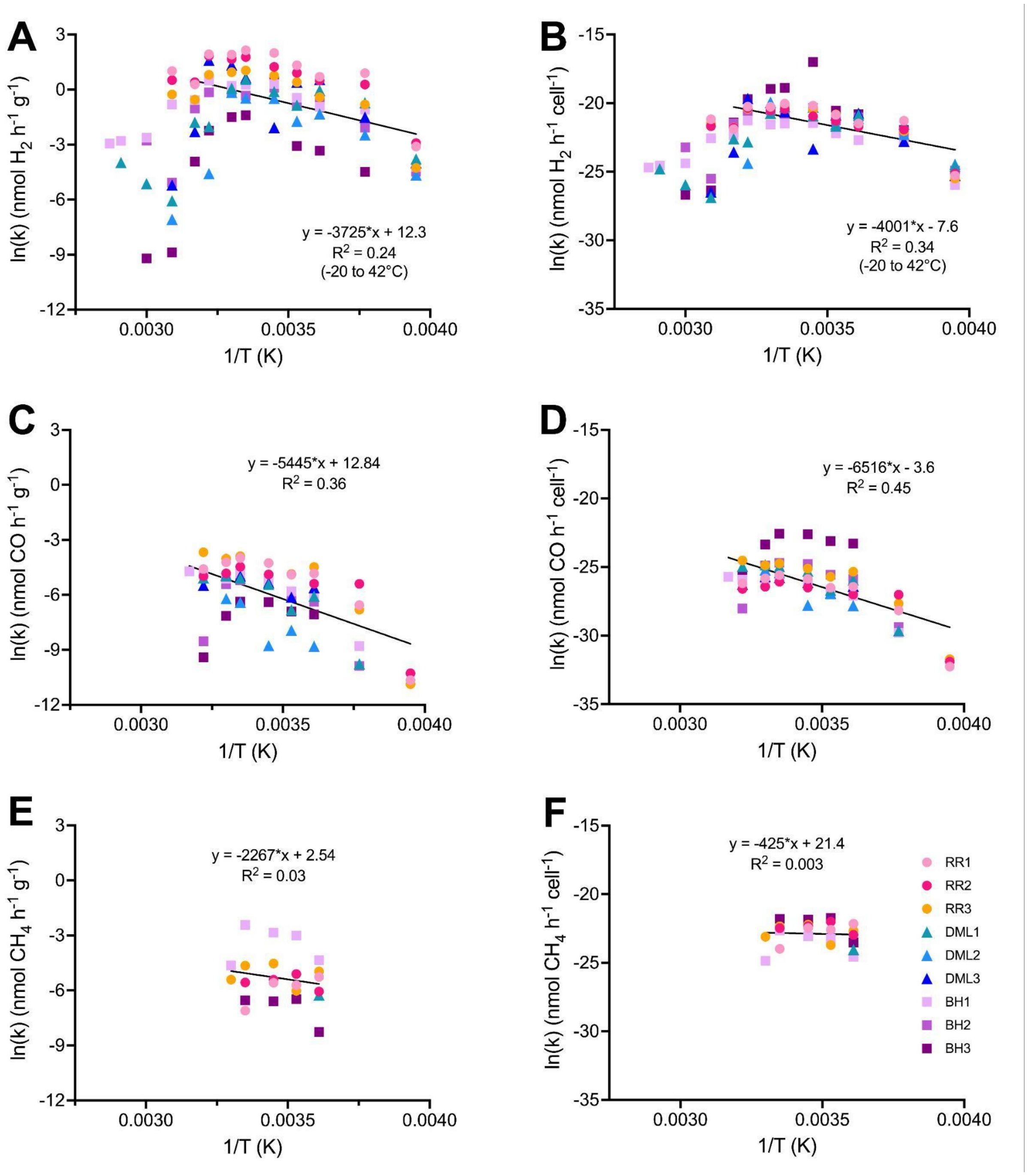

Figure S10. Arrhenius relationships fitted to bulk and cell-specific oxidation rates. Arrhenius plots of both bulk oxidation rates (nmol h^{-1} g^{-1}, **A,C,E**) and cell-specific oxidation rates (nmol h^{-1} $cell^{-1}$, **B,D,F**). The Arrhenius equation was fitted to all soils from -20 to 42°C and is displayed in black. RR, Robinson Ridge; DML, Dronning Maud Land; BH, Bunger Hills.

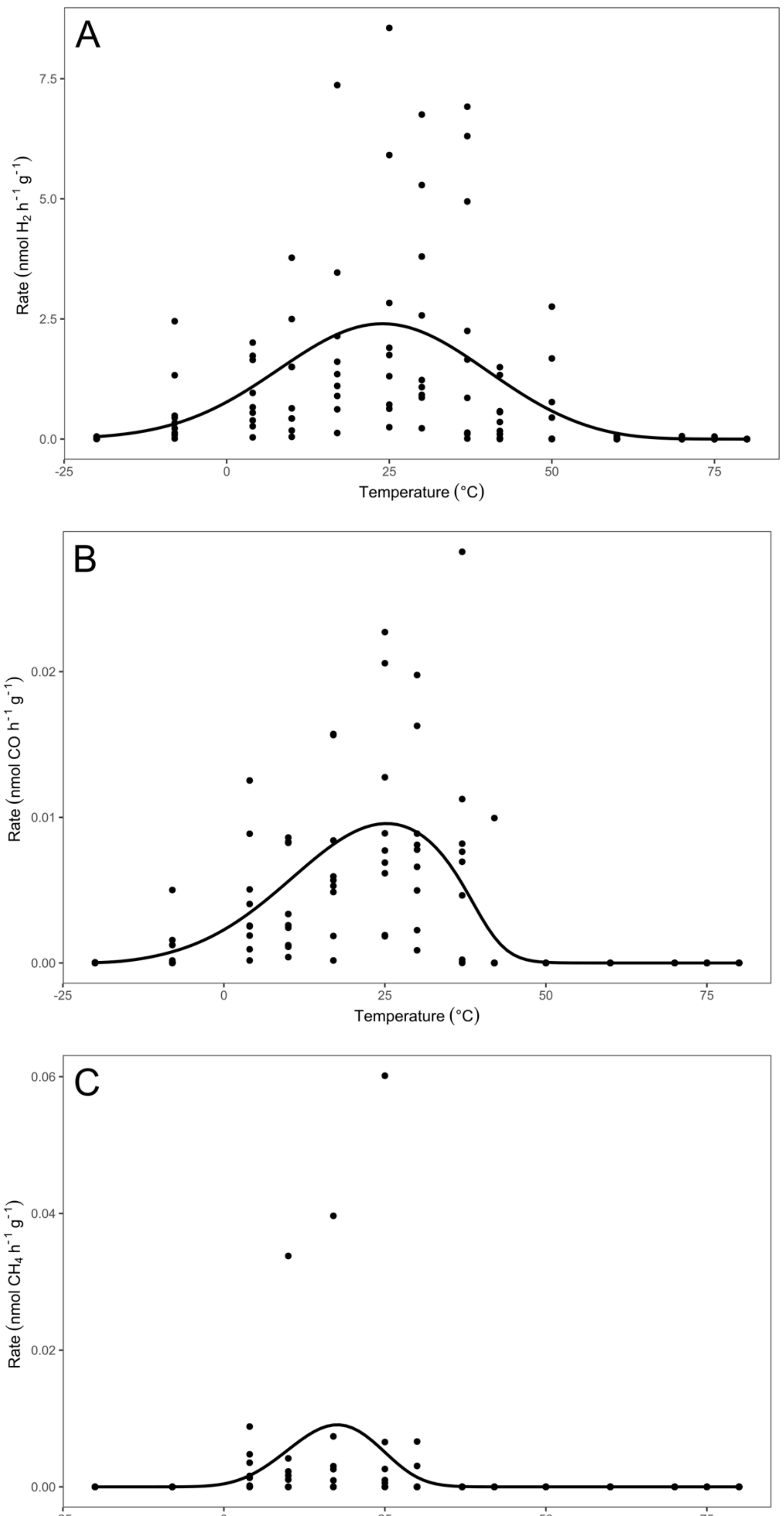

Figure S11. Relationships between temperature (°C) and rates of trace gas oxidation. (**A**) H_2, (**B**) CO, and (**C**) CH_4. For each temperature-rate relationship, the solid line represents the predicted relationship derived from the zero-inflated generalised linear model, using a Gamma distribution and a log-link function.
